# Anorexia nervosa, depression and suicidal thoughts among Chinese adolescents: a national school-based cross-sectional study

**DOI:** 10.1186/s12199-017-0639-2

**Published:** 2017-04-04

**Authors:** Qiguo Lian, Xiayun Zuo, Yanyan Mao, Shan Luo, Shucheng Zhang, Xiaowen Tu, Chaohua Lou, Weijin Zhou

**Affiliations:** 1 School of Public Health, Fudan University, Shanghai, China; 2 Key Lab. of Reproduction Regulation of NPFPC, SIPPR, IRD, Fudan University, 779 Laohumin Road, Shanghai, 200237 China; 3 West China Second University Hospital, Sichuan University, Chengdu, Sichuan China; 4 National Research Institute for Family Planning, Beijing, China

**Keywords:** Suicidal thoughts, Depression, Anorexia nervosa, Adolescents, China

## Abstract

**Background:**

Although there is much literature on adolescent suicide, combined effects of depression and anorexia nervosa on suicide were rarely investigated. The aims of this study are to examine the association between anorexia nervosa and suicidal thoughts and explore the interaction between anorexia nervosa and depression.

**Methods:**

This is a cross-sectional study, in the study, a sample of 8,746 Chinese adolescents was selected by multistage stratified method in 2012/2013 from 20 middle schools in 7 provinces across China Mainland. Multilevel logistic model was introduced to explore association between anorexia nervosa and suicidal thoughts. And subgroup analyses were conducted on participants with or without depression.

**Results:**

Multilevel logistic model revealed that demographic variables, including academic achievement, were not the predictive risk factors of suicidal thoughts. Those who suffered from worse severity of perceived anorexia nervosa were at increased risk of thinking about suicide. The interaction between depression and anorexia nervosa was significant, however, subgroup analyses showed that the associations were significant only among the adolescents without depression.

**Conclusions:**

Our results indicate that all levels of anorexia nervosa serve as predictable indicators of suicidal thoughts in Chinese adolescents, and the effects of anorexia nervosa are modified by depression status.

## Background

Suicide as a public health concern is largely preventable in adolescents [1, 2]. Adolescents are at high risk of suicide because they are particularly sensitive to environment during the stressful period of physical and cognitive development transitions [3]. There is some evidence to show that suicidal thoughts are becoming increasingly common in Asian youth [4–6]. Identifying subgroups of adolescents with suicidal thoughts could help guide suicide prevention initiatives, because existing studies indicate that suicidal ideation is the significant predictor of attempted suicide [7] and 60% of the suicide attempts emerge within the first year of the onset of suicidal thoughts [8].

It has been evidenced in many studies that anorexia nervosa is a serious psychiatric illness that is consistently associated with increased risk of suicide, especially in adolescents and young adults [9–13]. Some studies, however, suggested that completed suicide rates are not higher among individuals who are currently suffering from anorexia nervosa [14]. The results from another study confirmed that the null hypothesis, suicide rates, measured by officially recorded death certificates, are similar in the groups with or without anorexia nervosa [15]. However, most previous studies were based on clinical or community cohorts, thus the samples were small and focused on adults [9].

A growing body of literature shows that suicidal thoughts are associated with depression in adolescents [16–19]. Anorexia nervosa and major depression share common genetic and environmental risk factors [20]. Adolescents who suffered from anorexia nervosa have elevated risk of experiencing depression [20, 21]. There is much empirical literature on adolescent suicide, however, the interaction between depression and anorexia nervosa was rarely included in the models, and its influence on suicidal thoughts still remains unclear. To bridge this gap, the aims of this study are to evaluate the association between suicidal thoughts with anorexia nervosa among adolescents in China, and determine whether this association differed between the subgroups with or without depression.

## Methods

### Study sites and participants

This study was a school-based cross-sectional study, and conducted among 20 sampled middle schools in 7 provinces in China from 2012 to 2013. Multistage cluster sampling strategy was introduced to ensure a representative sample. First, given the heavily unbalanced development of China, Hebei and Inner Mongolia in the North China, Shandong in the East China, Heilongjiang in the Northeast China, Guangxi in the South China, Sichuan in the Southwest China and Shaanxi in the Northwest China that represented 6 administrative divisions were selected from 31 provinces in China. Second, one senior high school and one junior high school that represented the average education level were selected from the rural area and the urban area of one general socioeconomic status city in each selected province, respectively. Twenty schools were selected because some schools run both junior and senior high school programs. Finally, two classes were randomly selected from each grade at each school. All 9,312 students from those classes were recruited to the survey, and the response rate was 95.77% (8,746/9,312).

### Measures

The computer-assisted self-administered survey was conducted anonymously in school computer labs within 45 min. The content of the questionnaire included the history of suicidal thoughts, level of anorexia nervosa, depression status and demographic characteristics. All the items measuring suicidal thoughts, anorexia nervosa and depression were selected from the literature, and their reliability and validity were good [6, 22, 23].

### Suicidal thoughts

Suicidal thoughts were assessed as by one question: “Have you ever seriously thought about suicide? (1 = yes, 0 = no)” [6].

### Anorexia nervosa

The level of anorexia nervosa was measured by the question “Have you ever experienced the feeling of disgust by the sight of food)?” The answer is coded as “1 = never, 2 = occasionally, 3 = sometimes, 4 = often, 5 = always”. Items 4 and 5 were collapsed into a category of “often” [22].

### Depression

The sign of depression was evaluated by one question: “Have you ever experienced of feeling depressed / life is not worthwhile that lasts at least 2 weeks? (1 = yes, 0 = no)” [23].

### Demographic characteristics

The questionnaire included a wide variety of demographic information, including age, gender, grade, academic achievement, living arrangement, family socioeconomic status, relationship with parents and classmates, feelings about school, reactions to parent-child conflict and smoking history.

Relationship with parents and classmates, and feelings about school were graded as good, average and poor. Academic achievement and family socioeconomic status were rated as high, average and low. Smoking was defined by one question: “Have you ever tried smoking, even only one or two puffs? (1 = yes, 0 = no)” Personality trait was measured by the question” How do you react to parent-child conflict?” with possible responses of “1 = endure quietly under any conditions, 2 = endure quietly under most conditions, 3 = argue with parents under most conditions, 4 = argue with parents under all conditions”. Items 1 and 2 were collapsed into a category of “suffer in silence”, and items 3 and 4 were combined into a category of “argue with parents”.

### Statistical analysis

The prevalence of demographic characteristics and primary outcomes was presented by contingency tables. Odds ratios (ORs) and 95% confidential intervals (CIs) for the association between suicidal thoughts and anorexia nervosa were estimated by using logistic regression models with adjustment for potential confounders. Adolescents who never reported anorexia nervosa served as the reference group. Given that students are clustered within schools, the assumption of independence of the observations is violated because the residuals might be correlated within each cluster. We estimated multilevel logistic regression models for the hierarchical data, with students (level 1) nested within schools (level 2). The interactions of anorexia nervosa and depression were introduced on the basis of hierarchical model. Besides, subgroup analysis stratified by depression was performed. The significance was considered at *p* < 0.05. All the analyses were conducted with Stata/SE 14.2 [24].

## Results

### Demographic characteristics of participants

Of all the 8,746 participants, 50.39% were female and 49.53% were senior school students. The mean age was 15.38 ± 1.95 years. Descriptive statistics of the basic demographic characteristics are presented in Table 1. Most of the students (74.81%) lived with their two biological parents and 16.52% came from low socioeconomic status (SES) backgrounds. As to their academic achievements, 41.22% of the students were rated as good and 17.60% as below average. Less than 3% reported poor relationships with parents and classmates and 5% had bad feelings about school. More than 20% of the students tried smoke and the same proportion would argue with their parents when parent-child conflict occurs. 70% of the respondents suffered from anorexia, at least some level. 12.22% considered suicide seriously, and 22.32% struggled with depression.

**Table 1:** Characteristics of the Study Population (*N* = 8,746)

Variables	Number (%)
Gender	
Male	4,339(49.61)
Female	4,407(50.39)
Grade	
Junior school students	4,414(50.47)
Senior school students	4,332(49.53)
Smoking	
Yes	1,877(21.46)
No	6,869(78.54)
Academic achievement	
High	3,605(41.22)
Average	3,602(41.18)
Low	1,539(17.60)
Living arrangement	
Two biological parents	6,543(74.81)
Biological father or mother	921(10.53)
Others	1,282(14.66)
Family socioeconomic status	
High	472(5.40)
Average	6,829(78.08)
Low	1,445(16.52)
Relationship with parents	
Good	7,152(91.90)
Average	533(6.85)
Poor	97(1.25)
Relationship with classmates	
Good	5,255(60.08)
Average	3,266(37.34)
Poor	225(2.57)
Feelings about school	
Good	5,249(60.02)
Average	3,061(35.00)
Bad	436(4.99)
Reactions to parent-child conflict	
Suffer in silence	6,898(78.87)
Argue with parents	1,848(21.13)
Anorexia nervosa	
Never	2,538(29.02)
Occasionally	4,783(54.69)
Sometimes	1,169(13.37)
Often	256(2.93)
Suicidal thoughts	
Yes	1,069(12.22)
No	7,677(87.78)
Depression	
Yes	1,952(22.32)
No	6,794(77.68)

### Associations between suicidal thoughts and demographic characteristics

The associations between suicidal thoughts and demographic characteristics are presented in Table 2, by univariate and multivariate logistic models. As showed in univariate logistic models, almost all the demographic characteristics were significantly related to suicidal thoughts, including gender, grade, smoking, academic achievement, living arrangement, SES, relationships with parents and classmates, as well as feeling about school, strategies for parent-child conflict, anorexia and depression. In multivariate logistic models, suicidal thoughts were significantly correlated with all of the characteristics except academic achievement and living arrangement.

**Table 2:** Associations between suicidal thoughts and demographic characteristics

Variables	Univariate OR (95% CI)	*P* Value	Multivariate^a^ OR (95% CI)	*P* Value
Gender(female)	1.42(1.25, 1.62)	<0.001	1.89(1.59,2.23)	<0.001
Grade(Senior)	0.99(0.87, 1.12)	0.870	0.67(0.56,0.79)	<0.001
Smoking(Yes)	1.97(1.71,2.27)	<0.001	1.97(1.63,2.37)	<0.001
Academic achievement				
Good	1.00	-	1.00	-
Average	1.12(0.97, 1.30)	0.120	0.93(0.79,1.11)	0.450
Below average	1.64(1.38, 1.94)	<0.001	1.10(0.89,1.36)	0.390
Living arrangement				
Two biological parents	1.00	-	1.00	-
Biological father or mother	1.76(1.46, 2.12)	<0.001	1.18(0.82,1.70)	0.370
Others	1.53(1.29, 1.81)	<0.001	0.97(0.77,1.21)	0.790
Family socioeconomic status				
High	1.00	-	1.00	-
Average	0.69(0.53,0.89)	0.005	0.76(0.55,1.05)	0.100
Low	1.06(0.80,1.41)	0.680	0.89(0.62,1.28)	0.550
Relationship with parents				
Good	1.00	-	1.00	-
Average	2.45(1.97,3.05)	<0.001	1.61(1.26,2.05)	<0.001
Poor	3.95(2.55,6.11)	<0.001	1.84(1.12,3.03)	0.020
Relationship with classmates				
Good	1.00	-	1.00	-
Average	1.56(1.37,1.78)	<0.001	1.28(1.08,1.50)	0.003
Poor	3.59(2.65,4.86)	<0.001	1.79(1.23,2.63)	0.003
Feelings about school				
Good	1.00	-	1.00	-
Average	1.97(1.72,2.25)	<0.001	1.41(1.19,1.67)	<0.001
Bad	3.89(3.09,4.90)	<0.001	1.77(1.32,2.38)	<0.001
Anorexia nervosa				
Never	1.00	-	1.00	-
Occasionally	1.57(1.33,1.85)	<0.001	1.21(0.99,1.46)	0.050
Sometimes	2.21(1.79,2.72)	<0.001	1.25(0.97,1.61)	0.080
Often	3.99(2.92,5.44)	<0.001	1.94(1.32,2.85)	0.001
Strategies for conflict(Struggle)	1.57(1.34,1.81)	<0.001	1.34(1.12,1.59)	0.001
Depression(yes)	6.58(5.75,7.53)	<0.001	5.04(4.31,5.90)	<0.001

*CI* confidence interval, *OR* odds ratio

^a^Adjusted for potential confounders, including gender, grade, smoking, academic achievement, living arrangement, SES, relationship with parents and classmates, feeling about school, strategies for conflict and depression

### Association of anorexia, depression, and interactions with suicidal thoughts

As illustrated in Table 2, only the serious level of anorexia was related significantly to suicidal thoughts (OR 1.94; 95% CI 1.32-2.85) after adjustment of observed confounders. The adolescents with depression were at much higher risk of experiencing suicidal thoughts (OR 5.04; 95% CI 4.31-5.90) compared with their counterparts without depression. Moreover, significant interactions between anorexia and depression were observed in adjusted model (*P* < 0.05 on all levels, not shown in the table). After adjustment for the interactions, all levels of anorexia were related significantly to suicidal thoughts and worse anorexia was associated with higher probability of suicidal thoughts (Table 3). The association between depression and suicidal thoughts was much stronger (OR 7.25; 95% CI 5.18-10.14).

**Table 3:** Association of anorexia nervosa, depression and their interaction with suicidal thoughts

Variables		With depression(*n* = 1,952)^a^		Without depression(*n* = 6,794)^a^		Full model(*n* = 8,746)^b^	
		OR (95% CI)	*P* Value	OR (95% CI)	*P* Value	OR (95% CI)	*P* Value
All (*n* = 8,746)	Anorexia nervosa						
	Never(n = 2,538)	1.00	-	1.00	-	1.00	-
	Occasionally(n = 4,783)	0.96(0.71,1.28)	0.760	**1.38(1.07,1.79)**	**0.010**	**1.41(1.10,1.82)**	**0.008**
	Sometimes(n = 1,169)	0.93(0.65,1.33)	0.700	**1.53(1.08,2.18)**	**0.020**	**1.61(1.14,2.28)**	**0.007**
	Often(n = 256)	1.30(0.78,2.19)	0.320	**2.82(1.62,4.91)**	**<0.001**	**3.02(1.75,5.23)**	**<0.001**
	Depression(yes)	-	-	-	-	**7.25(5.18,10.14)**	**<0.001**
Male (*n* = 4,339)	Anorexia nervosa	With depression(*n* = 985)^a^		Without depression(*n* = 3,354)^a^		Full model(*n* = 4,339)^b^	
	Never(*n* = 1,375)	1.00	-	1.00	-	1.00	-
	Occasionally(*n* = 2,318)	1.10(0.73,1.67)	0.645	1.36(0.93,2.00)	0.114	1.26(0.96,1.67)	0.101
	Sometimes(*n* = 530)	0.82(0.47,1.43)	0.489	0.85(0.45,1.61)	0.622	0.92(0.61,1.38)	0.688
	Often(*n* = 116)	0.57(0.24,1.36)	0.207	**2.75(1.12,6.77)**	**0.027**	1.16(0.61,2.21)	0.652
	Depression(yes)	-	-	-	-	**5.21(4.11,6.61)**	**<0.001**
Female (*n* = 4,407)	Anorexia nervosa	With depression(*n* = 967)^a^		Without depression(*n* = 3,440)^a^		Full model(*n* = 4,407)^b^	
	Never(*n* = 1,163)	1.00	-	1.00	-	1.00	-
	Occasionally(*n* = 2,465)	0.84(0.55,1.28)	0.407	1.38(0.97,1.95)	0.073	1.18(0.90,1.53)	0.231
	Sometimes(*n* = 639)	0.96(0.59,1.57)	0.872	**2.08(1.33,3.24)**	**0.001**	**1.52(1.09,2.11)**	**0.013**
	Often(*n* = 140)	**2.17(1.05,4.48)**	**0.036**	**2.91(1.42,5.95)**	**0.003**	**2.67(1.63,4.39)**	**<0.001**
	Depression(yes)	-	-	-	-	**5.00(4.03,6.19)**	**<0.001**

*CI* confidence interval, *OR* odds ratio

^a^Adjusted for potential confounders listed in Table 2

^b^Adjusted for potential confounders listed in Table 2 and interactions between anorexia nervosa and depression

Results in bold indicate the associations were significant at 0.05 level

### Association between anorexia and suicidal thoughts, stratified by depression

We also performed subgroup analyses stratified by depression. After controlling for a broad range of potential confounders, there were no statistically significant associations between anorexia and suicidal thoughts among the adolescents with depression, as shown in Table 3. The associations, however, were significant at all levels of anorexia among the adolescents without depression. Those who reported worse severity of perceived anorexia had a higher risk of experiencing suicidal thoughts (OR = 1.38 at “occasionally” anorexia, OR = 1.53 at “sometimes” anorexia, and OR = 2.82 at “often” anorexia).

### Association between anorexia and suicidal thoughts, stratified by gender

Given than females may be more likely to suffer from anorexia nervosa, depression and suicidal thoughts than males, we performed gender-specific analysis (Table 3), and it returned a bit different results. The association between depression and suicidal thoughts was still strong (OR = 5.21 for males, OR = 5.00 for females). The associations were non-significant at all levels of anorexia among the males, and were only significant at severe levels of anorexia among the females (OR = 1.52 at “sometimes” anorexia, and OR = 2.67 at “often” anorexia). Females with severe levels of anorexia were more likely to report suicidal thoughts whether depression co-occurred or not, while only normal males with severest level of anorexia nervosa had elevated suicidal thoughts.

## Discussion

Our results indicate that all levels of anorexia nervosa serve as indicators of remarkable suicidal thoughts, which consists with the results of several previous researches [15, 25–28]. Few of Existing studies reported the dose-response relationship between levels of anorexia nervosa and suicidal thoughts. We assumed that the worse the anorexia nervosa, the higher the risk of suicidal thoughts, and vice versa. The findings confirmed our hypothesis and high level of anorexia nervosa was associated with an increased risk of suicidal thoughts. The greatest severity of anorexia nervosa was at the highest risk among three levels of anorexia nervosa for suicidal thoughts (OR 3.02, 95%CI 1.75-5.23).

To our knowledge, this is the first study with a national school-based sample in China that investigated the interaction between anorexia nervosa and depression and its impact on suicidal thoughts. Depression and anorexia nervosa symptoms often co-occur [19]. Depression may cause anorexia nervosa, and the condition, in turn, can also lead to depression. Both symptoms can trigger the birth of suicidal thoughts [15, 29]. Our findings suggest that depression might modify the association between anorexia nervosa and suicidal thoughts since the interaction between depression and anorexia nervosa was significant.

It has been demonstrated there is a strong association between suicidal thoughts and depression [29–32]. Our findings reveal an additive synergism between depression and anorexia nervosa in suicidal thoughts. However, in our study, anorexia nervosa predicts suicidal thoughts only occur in adolescents without depression, and the associations are gender specific, which indicates that depression and anorexia nervosas may share an etiological mechanism on suicidal thoughts. In the full model that controlling the potential confounders and interaction, depression makes a much greater contribution to increased risk of suicidal thoughts than anorexia nervosa. Another possible reason behind this counterintuitive finding is that depressed adolescents in this study may be under sampled and lead to selection bias [33]. Depressed adolescents were more than 2.75 times likely to withdraw from school compared with their peers without depression [34]. Besides, the students with anorexia nervosa are at greater risk of quitting school than general population, and hence the association between anorexia nervosa and suicidal thoughts is probably underestimated.

In most studies conducted in clinics, patients with anorexia nervosa are diagnosed by the WHO ICD-10 or DSM-IV [22]. However, in the large epidemiological studies, especially in school based studies, it’s unfeasible in practice. In current study, we used self-rated feeling of disgust toward food as a predictor of anorexia nervosa, given the evidence that anorexia nervosa is associated with elevated disgust sensitivity in young adults, especially in the domain of food [35]. It is noteworthy that there is a decrease in emotive responses to food with increasing age [36], which means we might overestimate the association between feeling of disgust toward food and anorexia nervosa among adolescents.

Similarly, we chose one self-rated question to identity adolescents with depression. Ultrashort screening instruments, such as one question used in our study, may detect, but not diagnose, depression. However, ultrashort screening instruments have advantages, especially in large national epidemiologic surveys, shorter instruments take less time and gain greater acceptance [37]. There is evidence that ultrashort screening instruments are as effective as longer screening instruments, such as the Center for Epidemiologic Depression Scale [37]. Patient Health Questionnaire (PHQ)-2 is very similar with one question used in our study. PHQ -2 asks two simple questions about mood and anhedonia, and has been found to be up to 74% sensitivity and 75% specificity in adolescents [38]. We think the ultrashort screening instrument in our study are as effective as PHQ-2, and further research is needed to be done.

Several limitations should be considered in interpreting our findings. First, the nature of cross-sectional design does not allow us to make causal inferences. Longitudinal studies are required to confirm the cause-effect relationships. Second, we did not assess characteristics of those who had completed suicide, which may introduce survivor bias. Last but not least, just like any other study with self-report measures, there is possibility of information bias. However, computer-assisted self-interviewing technology could create a sense of anonymity and minimize response bias [39].

## Conclusions

The present study suggests that anorexia nervosa increases the probability of suicidal thoughts in Chinese adolescents. More precisely, the worse the anorexia nervosa, the higher the risk of suicidal thoughts. Depression can modify the association. Subgroup analyses reveal that anorexia nervosa failed to predict suicidal thoughts in adolescents with depression. The causes of suicide are multi-factorial in origin involving a complex interplay of biological, social, cultural and psychological factors. More research is needed to explore the mechanisms behind. Nonetheless, our findings might shed light upon eating disorder and suicide prevention programs.

## Abbreviations

CI: Confidential interval
OR: Odds ratio
SES: Socioeconomic status

## References

[1] Hawton K, van Heeringen K (2009). Suicide. Lancet.

[2] Hawton K, Saunders KE, O'Connor RC (2012). Self-harm and suicide in adolescents. Lancet.

[3] Wolff J, Frazier EA, Esposito-Smythers C, Burke T, Sloan E, Spirito A (2013). Cognitive and social factors associated with NSSI and suicide attempts in psychiatrically hospitalized adolescents. J Abnorm Child Psychol.

[4] Lian Q, Zuo X, Lou C, Gao E, Cheng Y (2015). Sexual orientation and risk factors for suicidal ideation and suicide attempts: a multi-centre cross-sectional study in three Asian cities. J Epidemiol.

[5] Chen YY, Wu KC, Yousuf S, Yip PS (2012). Suicide in Asia: opportunities and challenges. Epidemiol Rev.

[6] Blum R, Sudhinaraset M, Emerson MR (2012). Youth at risk: suicidal thoughts and attempts in Vietnam, China, and Taiwan. J Adolesc Health.

[7] Nock MK, Borges G, Bromet EJ, Alonso J, Angermeyer M, Beautrais A, Bruffaerts R, Chiu WT, de Girolamo G, Gluzman S (2008). Cross-national prevalence and risk factors for suicidal ideation, plans and attempts. Br J Psychiatry.

[8] Badiee J, Moore DJ, Atkinson JH, Vaida F, Gerard M, Duarte NA, Franklin D, Gouaux B, McCutchan JA, Heaton RK (2012). Lifetime suicidal ideation and attempt is common among HIV+ individuals. J Affect Disord.

[9] Kostro K, Lerman JB, Attia E (2014). The current status of suicide and self-injury in eating disorders: a narrative review. J Eat Disord.

[10] Mekori E, Halevy L, Ziv SI, Moreno A, Enoch-Levy A, Weizman A, Stein D: Predictors of short-term outcome variables in hospitalised female adolescents with eating disorders. International Journal of Psychiatry in Clinical Practice. 2017;21:41–49. 10.1080/13651501.2016.1229794 27646309

[11] Pompili M, Mancinelli I, Girardi P, Ruberto A, Tatarelli R (2004). Suicide in anorexia nervosa: a meta-analysis. Int J Eat Disord.

[12] Wade TD, Fairweather-Schmidt AK, Zhu G, Martin NG: Does shared genetic risk contribute to the co-occurrence of eating disorders and suicidality? International Journal of Eating Disorders. 2015;48:684-91. 10.1002/eat.22421 25945699

[13] Ruuska J, Kaltiala-Heino R, Rantanen P, Koivisto AM (2005). Psychopathological distress predicts suicidal ideation and self-harm in adolescent eating disorder outpatients. Eur Child Adolesc Psychiatry.

[14] Guillaume S, Jaussent I, Olie E, Genty C, Bringer J, Courtet P, Schmidt U (2011). Characteristics of suicide attempts in anorexia and bulimia nervosa: a case-control study. PLoS One.

[15] Coren S, Hewitt PL (1998). Is anorexia nervosa associated with elevated rates of suicide?. Am J Public Health.

[16] Garcia-Alba C (2004). Anorexia and depression: depressive comorbidity in anorexic adolescents. Span J Psychol.

[17] Smith C, Steiner H (1992). Psychopathology in anorexia nervosa and depression. J Am Acad Child Adolesc Psychiatry.

[18] Kennedy SH, Kaplan AS, Garfinkel PE, Rockert W, Toner B, Abbey SE (1994). Depression in anorexia nervosa and bulimia nervosa: discriminating depressive symptoms and episodes. J Psychosom Res.

[19] Hendren RL (1983). Depression in anorexia nervosa. J Am Acad Child Psychiatry.

[20] Koutek J, Kocourkova J, Dudova I (2016). Suicidal behavior and self-harm in girls with eating disorders. Neuropsychiatr Dis Treat.

[21] Wade TD, Bulik CM, Neale M, Kendler KS (2000). Anorexia nervosa and major depression: shared genetic and environmental risk factors. Am J Psychiatry.

[22] Aharoni R, Hertz MM (2012). Disgust sensitivity and anorexia nervosa. Eur Eat Disord Rev.

[23] Vahter L, Kreegipuu M, Talvik T, Gross-Paju K (2007). One question as a screening instrument for depression in people with multiple sclerosis. Clin Rehabil.

[24] StataCorp (2016). Stata Statistical Software: Release 14.2.

[25] Signorini A, De Filippo E, Panico S, De Caprio C, Pasanisi F, Contaldo F (2007). Long-term mortality in anorexia nervosa: a report after an 8-year follow-up and a review of the most recent literature. Eur J Clin Nutr.

[26] Latzer Y, Hochdorf Z (2005). Dying to be thin: attachment to death in anorexia nervosa. Sci World J.

[27] Pompili M, Girardi P, Tatarelli G, Ruberto A, Tatarelli R (2006). Suicide and attempted suicide in eating disorders, obesity and weight-image concern. Eat Behav.

[28] Sachs KV, Harnke B, Mehler PS, Krantz MJ (2016). Cardiovascular complications of anorexia nervosa: a systematic review. Int J Eat Disord.

[29] Lamis DA, Ballard ED, May AM, Dvorak RD (2016). Depressive symptoms and suicidal ideation in college students: the mediating and moderating roles of hopelessness, alcohol problems, and social support. J Clin Psychol.

[30] Kaltiala-Heino R, Rimpelä M, Marttunen M, Rimpelä A, Rantanen P (1999). Bullying, depression, and suicidal ideation in Finnish adolescents: school survey. BMJ.

[31] Klomek AB, Sourander A, Kumpulainen K, Piha J, Tamminen T, Moilanen I, Almqvist F, Gould MS (2008). Childhood bullying as a risk for later depression and suicidal ideation among Finnish males. J Affect Disord.

[32] Joffe BI, Van Lieshout RJ, Duncan L, Boyle MH (2014). Suicidal ideation and behavior in adolescents aged 12-16 years: a 17-year follow-up. Suicide Life Threat Behav.

[33] Hong L, Guo L, Wu H, Li P, Xu Y, Gao X, Deng J, Huang G, Huang J, Lu C (2016). Bullying, depression, and suicidal ideation among adolescents in the fujian province of china: a cross-sectional study. Medicine (Baltimore).

[34] Quiroga CV, Janosz M, Lyons JS, Morin AJS (2012). Grade retention and seventh-grade depression symptoms in the course of school dropout among high-risk adolescents. Psychology.

[35] Troop NA, Murphy F, Bramon E, Treasure JL (2000). Disgust sensitivity in eating disorders: a preliminary investigation. Int J Eat Disord.

[36] McNamara C, Hay P, Katsikitis M, Chur-Hansen A (2008). Emotional responses to food, body dissatisfaction and other eating disorder features in children, adolescents and young adults. Appetite.

[37] Choi SKY, Boyle E, Burchell AN, Gardner S, Collins E, Grootendorst P, Rourke SB, OCS Group (2015). Validation of six short and ultra-short screening instruments for depression for people living with HIV in Ontario: results from the Ontario HIV treatment network cohort study. PLOS One.

[38] Richardson LP, Rockhill C, Russo JE, Grossman DC, Richards J, McCarty C, McCauley E, Katon W (2010). Evaluation of the PHQ-2 as a brief screen for detecting major depression among adolescents. Pediatrics.

[39] Le LC, Blum RW, Magnani R, Hewett PC, Do HM (2006). A pilot of audio computer-assisted self-interview for youth reproductive health research in Vietnam. J Adolesc Health.

